# Phase Diagram and Transformations of Iron Pentacarbonyl to nm Layered Hematite and Carbon-Oxygen Polymer under Pressure

**DOI:** 10.1038/srep15139

**Published:** 2015-10-12

**Authors:** Young Jay Ryu, Minseob Kim, Choong-Shik Yoo

**Affiliations:** 1 Materials Science and Engineering, Department of Chemistry and Institute for Shock Physics, Washington State University, Pullman, Washington 99164.

## Abstract

We present the phase diagram of Fe(CO)_5_, consisting of three molecular polymorphs (phase I, II and III) and an extended polymeric phase that can be recovered at ambient condition. The phase diagram indicates a limited stability of Fe(CO)_5_ within a pressure-temperature dome formed below the liquid- phase II- polymer triple point at 4.2 GPa and 580 K. The limited stability, in turn, signifies the temperature-induced weakening of Fe-CO back bonds, which eventually leads to the dissociation of Fe-CO at the onset of the polymerization of CO. The recovered polymer is a composite of novel nm-lamellar layers of crystalline hematite Fe_2_O_3_ and amorphous carbon-oxygen polymers. These results, therefore, demonstrate the synthesis of carbon-oxygen polymer by compressing Fe(CO)_5_, which advocates a novel synthetic route to develop atomistic composite materials by compressing organometallic compounds.

The ability to modify chemical bonding in molecular systems by means of high-pressure and high-temperature opens new opportunities for synthesis of novel materials with unique properties desirable for energy applications. The application of thermo-mechanical energy (achieved by large compression at high pressure) comparable to that of chemical bond energies, for example, converts molecular solids into nonmolecular extended solids in high chemical energy states^1^ Importantly, these extended solids, when made of low Z elements in dense three-dimensional (*3D*) network structures (e.g. diamond and cubic-BN) exhibit fascinating thermal, mechanical and electro-optical properties such as high energy density, superhardness, superconductivity, magnetic correlation, and nonlinear optical properties^2^,^3^,^4^,^5^,^6^,^7^ While ubiquitous at high pressures, these low Z extended structures become highly metastable at ambient conditions. Hence, developing novel low Z extended solids for ambient uses poses great scientific and technological challenges.

Organometallic compounds, considering as metallic species in low Z C/H/N/O molecules, can provide intriguing opportunities, not only to tune the reaction parameters critical to the synthesis of high energy density extended solids, but also to produce monolithic low Z extended solids with functional transition metal atoms spread homogeneously over the host low Z lattice. The nature of metal-organic (C/N/O/H) bonds, for example, varies greatly depending on the metal^8^,^9^,^10^,^11^, forming a variety of bonds. They include highly ionic bonds (e.g., alkali metals such as Na, K, Rb, and Cs), polar multi-centered (Li, Be, B, Mg, and Al), directional σ and δ (Ti, Fe, Ni, and Pt), highly covalent σ (Si, P and Cu), and mixed δ- and *f*-bonds (lanthanides and actinides) with a large number (6 to 12) of highly ionic ligands. The polarity of metal-organic bonds and the size of metal ions influence the bond energy and structure of organometallic compounds. This variety in electron polarity, structure, strength, and bond energy of metal-carbon bonds, as well as the catalytic effect of metal species, can give rise to different reaction pathways to low Z extended network structures at moderate pressures, which can be recovered at ambient pressure and exhibit the functional properties of doped metals such as electric conductivity or magnetism.

Relatively weak metal-ligand bonds can thermally or photochemically decompose to metallic ions (or even stable radicals) and unsaturated ligands such as CO, CN, NO, and C_2_H_4_^12^,^13^,^14^. These ligands, on the other hand, can easily polymerize by themselves into network structures at moderate pressure (1–10 GPa)^2^,^15^,^16^,^17^,^18^. Yet, the metal species can serve as catalysts for the reactions as well as stabilizers for the products. This in turn can result in a homogeneous distribution of metals in low-Z extended solids. Such an incorporation of metal species in an atomistic scale differs from conventional reactive metal-polymer composites^19^,^20^ that are highly heterogeneous and often subjected to chemical and mechanical deterioration. The method can also be used for incorporating a wider range of metal species including highly reactive *f*-electron metals (lanthanides and actinides). Potentially advanced extended structures that are expected to form from these organometallic compounds under pressures include three-dimensional networks of interlinked chains, small-metal-intercalated layers, and spherical large-metallocenes^21^,^22^,^23^. Moreover, the extended organometallic compounds especially of magnetic *d/f*-transition metals can exhibit novel optical and electronic properties such as photochemical selectivity, ferroelectric and magnetic properties, and metallic and even superconductivities, all of which can be used to control and functionalize the extended solids.

The present study is to demonstrate the synthesis of extended carbon-oxygen polymer by compressing iron pentacarbonyl, Fe(CO)_5_. A critical step is to break iron-carbonyl bonds and expose catalytic iron sites to unsaturated carbonyl ligands at the onset of CO polymerization. Fortunately, most metal-ligand bonds are thermodynamically weak—certainly with respect to strong covalent bonds in unsaturated ligands—that often undergo homolytic decomposition under moderate heating or UV/VIS radiation. In fact, the organometallic compounds of *3d* metals are even thermolabile producing stable metal radicals: *e.g.,* Fe(CO)_5_ → Fe*+5CO^24^. Unsaturated CO, on the other hand, is known to polymerize under moderate pressures (~5–6 GPa) to polymeric CO (poly-CO) in a *3D* network structure^25^,^26^,^27^,^28^. Poly-CO is likely a high energy density solid, yet is highly metastable at ambient condition^2^,^25^,^26^,^27^. Thus, it is expected that the presence of metal atoms (or radicals) can passivate daggling bonds in the *3D* network and stabilize poly-CO at ambient conditions. Furthermore, the magnetic *3d-*transition metal iron may provide an added benefit to further development of a functional extended solid such as a super paramagnet.

In this study, we have investigated the physical and chemical transformations of Fe(CO)_5_ over a broad range of pressures and temperatures. The main results can be summarized into the phase diagram of Fe(CO)_5_ in Fig. 1, showing a series of phase transitions and polymerization to a novel nm-lamellar layer composite of crystalline hematite and amorphous carbon-oxygen polymer that, unlike poly-CO, is stable at ambient condition.

Iron pentacarbonyl undergoes a series of phase transitions under high pressures, as evident from its pressure-induced changes in visual appearance (Fig. 1 inset images) and the spectral Raman changes (Fig. 2). Iron pentacarbonyl is yellowish liquid at the ambient condition, which solidifies to phase I at around 0.2 GPa, then transforms to phase II at 1.0 GPa and phase III at 4.8 GPa. Phase I and II form relatively large crystalline grains, whereas phase III forms interesting striped crystals. Phase III is then stable over a large pressure range to 15.5 GPa, above which it chemically transforms into a polymeric product. The polymeric product can be recovered at ambient conditions, and the recovered product shows a lustrous surface appearance and is non-hygroscopic and chemical stable at ambient condition. These visual appearance changes of Fe(CO)_5_ also accompany the spectral Raman changes as shown in Fig. 2.

Following the previous vibrational mode assignments^29^,^30^, the observed Raman peaks of Fe(CO)_5_ can be characterized into three groups, as summarized in Table S1: (i) the C ≡ O stretching modes, ν_s_(CO), in the spectral region between 1800 and 2200 cm^−1^, (ii) the Fe-CO stretching modes, ν_s_(Fe-CO), and Fe-C ≡ O bending modes, ν_b_(Fe-C ≡ O), between 350 to 650 cm^−1^, and (iii) the C-Fe-C bending modes, ν_b_(C-Fe-C), below 300 cm^−1^. Fe(CO)_5_ has a bipyramidal structure with an Fe atom at the center and five carbonyls attached to the central Fe: three on the equatorial plane and two on the axial direction^31^. Therefore, the molecule belongs to the D_3h_ point group and has fundamental stretching vibrational modes of 2A_1_′ + A_2_′′ + E′ for both the Fe-C and C ≡ O bonds. Of these modes, the selection rule permits the A_2_′′ and E′ modes to be IR active and the totally symmetric 2A_1_′ and doubly degenerated (E′) Raman active, as observed in Fig. 2.

The phase transitions are apparent from the pressure-induced spectral changes in the Raman spectra of Fe(CO)_5_ along various isotherms, as illustrated in Fig. 2. The presence of liquid Fe(CO)_5_ is most evident from two broad ν_s_(CO) modes at around 2000 cm^−1^ and one broad ν_b_(C-Fe-C) at around 120 cm^−1^. Upon the solidification, each of these broad features splits into three sharp peaks. The phase I to II transition further splits the ν_b_(C-Fe-C) modes into 6–7 peaks, while the ν_s_(CO) peaks shift abruptly at the transition pressure (see Fig. 2). The phase II to III transition is, on the other hand, most evident from further splitting of ν_b_(C-Fe-C) into two groups of multiplets as well as from the emergence of two new vibrational modes at ~70 and 2010 cm^−1^ (see Fig. 2b). Upon the polymerization above 15.5 GPa, all sharp Raman features of Fe(CO)_5_ disappear, while a new broad band appears at ~1600 cm^−1^ (also in Fig. 3a). The emergence of this new broad band signifies the formation of conjugated C = O/C = C double bonds in a graphite-like layer structure and analogous to those observed in poly-CO. Note that there are small features at around 2000 cm^−1^ at 15.2 GPa and 300 K in Fig. 2a, which is absent at high temperatures. The pressure dependences of these features are similar to those of unreacted Fe(CO)_5_, as shown in Fig. 2b. Thus, we attribute these to untransformed Fe(CO)_5_. In fact, the polymerization occurs over a large pressure range, starting from 15 GPa to often as high as 20 GPa at ambient temperature. These spectral changes occur irreversibly upon compression.

Similar spectral changes are associated with the phase transitions at high temperatures. It is important to note that the polymerization occurs from different phases depending on the temperature; at 300 K from phase III, at 573 K from phase II, and at 623 K directly from liquid Fe(CO)_5_. Despite the thermal-path difference in temperature, we found no difference in the Raman spectra of recovered products, as illustrated in Fig. 3a.

The recovered products show an interesting layered morphology, as shown in Fig. 3b,c. The SEM image in Fig. 3b clearly shows a lustrous surface, similar to the optical image in Fig. 1, which can be peeled off as a thin layer. The layer structure of the product is more evident from the TEM image in Fig. 3c, showing a novel nm-lamellar layer structure. The layer thickness is estimated to be ~8 nm based on the x-ray diffraction data (see Fig. 4).

Raman spectra of the recovered products also support the layer structure and indicate a composite nature of the products, as shown in Fig. 3a. For example, the Raman spectrum obtained from the shinny surface layer shows a broad vibrational feature peaking at around 1600 cm^−1^, which likely arises from the C = C/C = O stretching mode in a 2D layer structure such as the graphite ν_s_(C = C) vibron at ~1600 cm^−1^^32^,^33^. The dull inside layer, on the other hand, shows a very different Raman spectrum, which consists of strong peaks at 1300 cm^−1^ and several peaks between 150 and 650 cm^−1^. Surprisingly, this Raman spectrum is nearly perfect match to that of Fe_2_O_3_ hematite, as compared in Fig. 3a^34^. Note that these Raman characteristics are observed independent of the temperature where it was synthesized, as expected from the phase diagram in Fig. 1. Note that there is no Raman feature of hematite observed *in-situ* at high pressures. This is likely due to high reflectivity of the surface layer, hampering the Raman scattering from the inside hematite layer.

To validate the hematite structure, we have obtained the x-ray diffraction pattern of the products at 20 GPa. A high quality powder diffraction pattern was obtained, which can be easily indexed to the hematite structure in a *R-3c* space group^35^,^36^. Figure 4 shows the observed (‘x’ symbols), the Rietveld refined (black line), and the difference (blue line) diffraction patterns. The refinement was based on the *R-3c* structure. The calculated peak positions for the hkl (labeled) lattice planes of the *R-3c* structure are marked as short vertical lines. The small intensity mismatch between the observed and refined diffraction patterns at the feet of the (104) peak at 2θ = 7.8° (*d* ~ 2.8 Å) and the (113) peak at 10.1° (*d* ~ 2.1 Å) can be easily understood in terms of the contribution from the (002) and (101) reflections of graphite-like layered amorphous carbon (marked as green lines) or carbon oxide products at ~20 GPa^37^. The best refinement of the observed data results in the lattice parameters: a = 4.574(2) Å, c = 14.356(2) Å, and ρ = 6.116 g/cm^3^. This result is, indeed, well compared with the previously reported data of pure α-Fe_2_O_3_ hematite at 19 GPa^35^,^36^, as summarized in Table S2.

It is important to note that all observed diffraction peaks are relatively broad, supporting the presence of nm-layered amorphous carbon oxides. In fact, the average crystallite size of hematite is estimated to be about 8.1 nm, based on the observed diffraction bandwidth D and the Scherrer equation^38^, 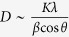, where *K* is the shape factor, used typical value of 0.89, β is line broadening, and θ is the Bragg angle.

The crystal structure of Fe_2_O_3_ hematite in polymeric CO mixture can be considered as a layer structure made of corner-sharing highly distorted trigonal pyramid. In this structure (summarized in Table S2), iron atoms are at 12c(0,0,z) with z = 0.854, three-fold coordinated with oxygen atoms at 18e(x, 0, 1/4) with x = 0.326, resulting in the nearest, intra-layer iron-oxygen bond distance at *d*_Fe-O1_ = 1.790 Å and the nearest inter-layer Fe-Fe distance of *d*_Fe-Fe2_ = 2.957 Å. The second nearest, inter-layer iron-oxygen distance is, on the other hand, at *d*_Fe-O2_ = 2.103 Å—substantially longer than the nearest one, *d*_Fe-O1_ = 1.790 Å, signifying the layer structure. As a result, the present Fe_2_O_3_ structure indicates a highly distorted nature of corner-sharing FeO_6_ octahedra. A similar distortion is also present in pure hematite, α-Fe_2_O_3_^35^,^36^, but to a substantially smaller degree; *d*_Fe-O1_ = 1.905 Å and *d*_Fe-O2_ = 2.132 Å.

It is interesting to note that the refined volume of Fe_2_O_3_ in carbon-oxygen polymer at 20 GPa is similar to that of pure hematite at 46 GPa^36^. Considering a continuous reduction of the c/a ratio of pure α-Fe_2_O_3_ under pressure (2.74 at ambient pressure to 2.64 at 60 GPa) and a significant larger c/a ratio (3.14) of the present Fe_2_O_3_, we conjecture that extra carbon or oxygen atoms may be present in the interstitial sites between two double layers of the *ab*-plane. This assumes the additional carbon and oxygen atoms to be produced during the pressure-induced polymerization from Fe(CO)_5_ to Fe_2_O_3_ and carbon-oxygen polymer—which is likely. In fact, ~5% larger interlayer distance (2.96 Å) of the present Fe_2_O_3_ at 20 GPa than that (2.811 Å) of α-Fe_2_O_3_ at 19 GPa supports this conjecture. The a and b-axes, on the other hand, are smaller than those of pure hematite. This can be understood in terms of a mixed octahedral structure of Fe_2_O_3_ (hematite): the face-shared octahedral along the c-axis and the corner-shared octahedra along the a- and b-axes. The interstitials are mostly located at the top and bottom of the face-shared octahedral layers. As such, as extra carbon and/or oxygen atoms are filled in the interstitials, it is likely that the c-axis increases, whereas the a and b-axes decrease—analogous to the behavior of zero thermal expansion materials^39^. As a result, the c/a ratio (3.14) of the present Fe_2_O_3_ becomes nearly twice of the closed packed value (1.63), which seems to explain the observed higher density of the present Fe_2_O_3_ (ρ = 6.117 g/cm^3^) than pure hematite (ρ = 5.548 g/cm^3^) at 19 GPa.

In conclusions, the present results provides constraints for the phase diagram of Fe(CO)_5_ shown in Fig. 1, highlighting several important facts regarding Fe(CO)_5_ and its polymerization. (i) Fe(CO)_5_ is stable only within the limited pressure-temperature range (the blue colored dome in Fig. 1) below the liquid- phase II- polymer triple point at 4.2 GPa and 580 K. (ii) Above the dome area (the red colored region), Fe(CO)_5_ chemically transforms to an atomistic composite of Fe_2_O_3_ and carbon-oxygen polymer; (iii) The polymerization can directly occur from liquid to the same product at ~4 GPa above ~600 K. The extrapolation of the liquid-polymer transition line yields the transition at around 6–7 GPa at 300 K, the pressure range where pure CO polymerizes. (iv) The enhanced stability at ambient temperature indicates the enhanced stability of Fe-CO back bonds under pressures. (v) Phase I is stable in narrow region near the melt temperatures and its structure is same as the previously determined C2/c at low temperature (see Table S3)^40^. Finally, the present results demonstrate the synthesis of carbon-oxygen polymer by compressing Fe(CO)_5_ and suggest a new synthetic route to develop transition metal bearing high energy density solids by compressing organometallic compounds.

## Methods

The present study was based on a large number of high-pressure experiments, providing a consistent and reproducible set of results. Iron pentacarbonyl (99.9%, Aldrich) was loaded into He-gas driven membrane diamond anvil cells (DAC) in a dry box flushed with inert Ar gas. Type IA diamond anvils were used with a flat culet size of 0.5 mm. A rhenium gasket was pre-indented to 0.08 mm thick, and a small hole of 0.2 mm in diameter was drilled using an electric-discharge micro-drilling machine. The sample was loaded together with a few small particles of Ruby for pressure measurements^41^. For high temperature experiments, DAC was externally heated using a band heater (Omega Co.). The temperature of the sample was measured within an accuracy of 5 degrees, using the K-type thermocouple (Omega Co.) at the back and the side of diamond anvil near the sample.

Raman spectroscopy was used to determine the pressure-induced phase and chemical transformations in a back scattering geometry, using a primary 514.5 nm excitation line from an Ar-ion laser (Spectra-Physics) coupled with a liquid-nitrogen cooled, back-illuminated CCD detector and a 0.5 m spectrograph with a 1800 line/mm holographic grating. The system is operated in a back scattering geometry using a holographic beamspitter (95% refraction at 514.5 nm, Kaiser Optics), a Raman notch filter (OD > 5, Kaiser Optics) and a long-working distance objective lens (20x, 32 mm WD, Edmund). It is also coupled with a pair of confocal lens and a 2D slit, providing a spatial resolution of 5 μm along the vertical axis of the sample and 3 μm on the horizontal plane. With a typical slit opening of 10 μm, the system yields the spectral resolution better than ~0.5 cm^−1^. Because Fe(CO)_5_ is highly photosensitive, we used a minimum level of laser power (less than 10 mW) to obtain Raman spectra of the sample, especially at low pressures below 1.5 GPa for liquid and phase I.

The recovered sample was characterized by a scanning electron microscope (SEM, FEI Quanta 200F) and a transmission electron microscope (TEM, FEI Tecnai G20 T-20 Twin) at the Washington State University’s Franceschi Electron Microscopy and Image Center.

For powder x-ray diffraction studies, we used a micro-focused monochromatic x-ray beam (λ = 0.3738 Å) from the HPCAT beamline (16IDB) at the Advanced Photon Source. Fe(CO)_5_ polymorphs typically result in highly preferably oriented crystals apparent from the spotty patterns with large variations in the peak intensities and its appearance. This makes difficult to determine the structure of Fe(CO)_5_ polymorphs. However, upon the polymerization the sample becomes highly polycrystalline and develops well-developed Debye-Scherr’s diffraction rings, which can be analyzed using the Fit2D program^42^.

The crystal structure was determined using DICVOL^43^ and the proposed space group was selected based on the systematic absence of the observed diffraction peaks. The detailed structural information of hematite was obtained by performing a full-scale Rietveld refinement. In this refinement, the scale factor, lattice parameters, zero shift, background, and peak width parameters, u, v, and w were all determined. The background parameters were not further refined after subtracted properly from the raw data. The atomic coordinates of Fe(0, 0, z) (12c) and O(x, 0, 0.25) (18e) were further refined with the Debye-Waller factors. In order to obtained the best fitting result, we introduced preferred orientation on the (104) peak. The refined intensity was in a good agreement with the experiment data with reduced χ^2^ = 0.319.

The fitting of feet of the asymmetric peaks at ~8°, ~10.3°, and ~11°, however, were not improved by including additional asymmetric fitting function and microstain factors. Such asymmetric tail was fit by adding an amorphous graphite phase^37^, which is determined as a hexagonal structure of a = 2.407 Å and c = 5.620 Å. Because the composition of amorphous carbon is not know in this study, we fitted the additional phase with Le Bail intensity fitting method^44^.

To determine the structure of unreacted Fe(CO)_5_ polymorphs, we employed a single crystal x-ray diffraction method using the ALS beamline (12.2.2) with λ = 0.3548 Å. A large single crystal of phase I was grown from the melt, to determine the crystal structure of phase I. In order to collect a large number of reflections, a modified Boehler–Almax-type DAC and a pair of BN seats were used. The DAC has a large opening angle to collect the data over an angular range from −45° to 45° and a Perkin-Elmer amorphous silicon flat-panel detector was used. Data was scanned three times per second per 0.25° rotation, and the total of 1874 reflections were used for the structure analysis. The peak indexing, determination and intensity integration was done by the APEX2 software. The structure of phase I was solved with a monoclinic space group C2/c and the structure refinement was performed using SHELXL^45^. The results are summarized in Table S3. However, the quality of single crystal becomes rapidly deteriorates upon the phase transition, hampering further determination of the crystal structure of phase II and III.

## Additional Information

**How to cite this article**: Ryu, Y. J. *et al.* Phase Diagram and Transformations of Iron Pentacarbonyl to nm Layered Hematite and Carbon-Oxygen Polymer under Pressure. *Sci. Rep.* **5**, 15139; doi: 10.1038/srep15139 (2015).

## Supplementary Material

Supplementary Information

## Figures and Tables

**Figure 1:**
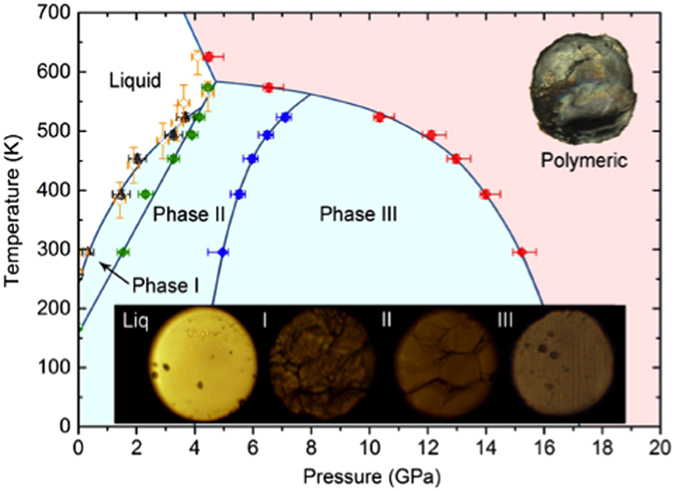
Phase diagram of Fe(CO)_5_, consisting of liquid, three molecular polymorphs (phase I, II, III), and a polymeric product. Note that unreacted Fe(CO)_5_ is stable within a limited pressure-temperature range (the blue-colored area), reflecting the weakness of Fe-CO back bonds with increasing temperatures. (**Inset**) Optical microscopic images of the sample at various pressures, showing the visual appearance to Fe(CO)_5_ polymorphs and a lustrous surface of the polymeric product coming from a graphite-like *2D* carbon-oxygen polymer. The open and close symbols signify the transformation points obtained along the isothermal compression and the isobaric heating, respectively; whereas, the color of the symbol represents the end phase of the transformation: liquid in yellow, red in polymer, phase I in black, II in green, and III in blue.

**Figure 2:**
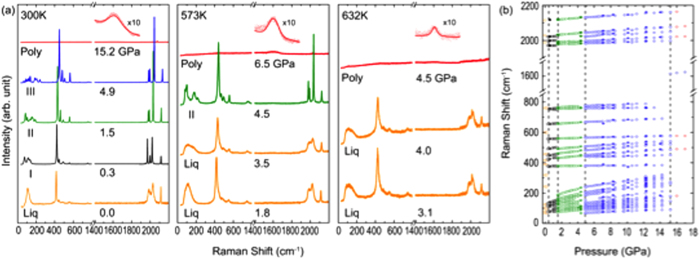
(**a**) Raman spectra of Fe(CO)_5_ at various pressure-temperature conditions, showing the pressure-induced phase transitions along several isotherms: liquid → phase I → phase II → phase III → polymer at 300 K, liquid → phase II → polymer at 573 K, and liquid → polymer at 623 K. (**b**) The pressure dependent Raman peak shifts at ambient temperature, showing the onset pressures of the phase transitions. The colors of the symbols signify the different end phases of transformations: orange for liquid, black for phase I, green for phase II, blue for phase III, and red for polymer. The open and closed symbols signify the data taken during the pressure uploading and downloading, respectively.

**Figure 3:**
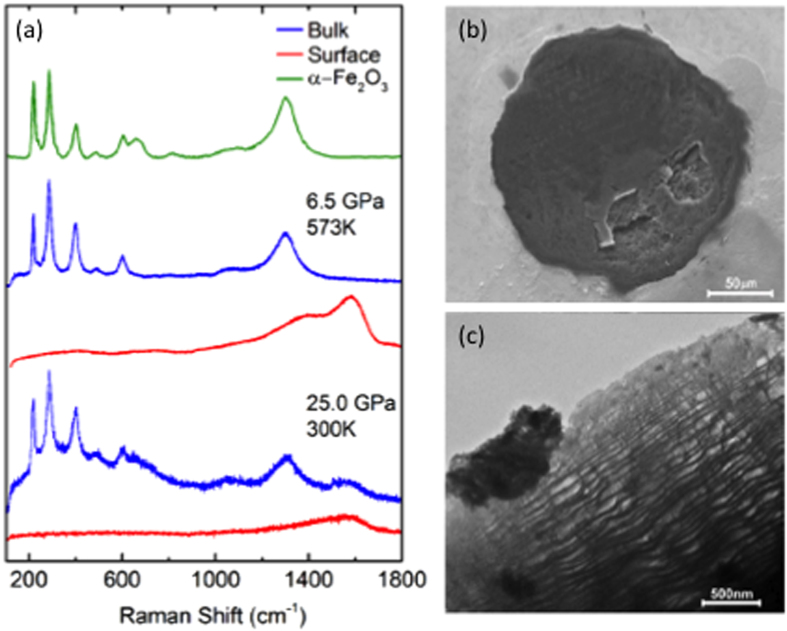
(**a**) Raman spectra obtained from the shinny surface (the red spectra) and the dark inside (the blue) of the recovered sample from 25 GPa and 300 K and 10 GPa and 600 K, plotting together with the Raman spectrum of hematite Fe_2_O_3_ (the green) previously obtained^34^ for comparison. The broad peak at around 1600 cm^−1^ of the surface Raman spectra indicates that it is made of a graphite-like *2D*, *sp*^*2*^ hybridized carbon oxides layer. (**b**) SEM and (**c**) TEM images of the recovered products, showing a nm-lamellar layer structure made of amorphous graphite-like carbon oxide layers (the surface in SEM or the light area in TEM) and crystalline iron oxide layers (the inside in SEM or the dark area in TEM).

**Figure 4:**
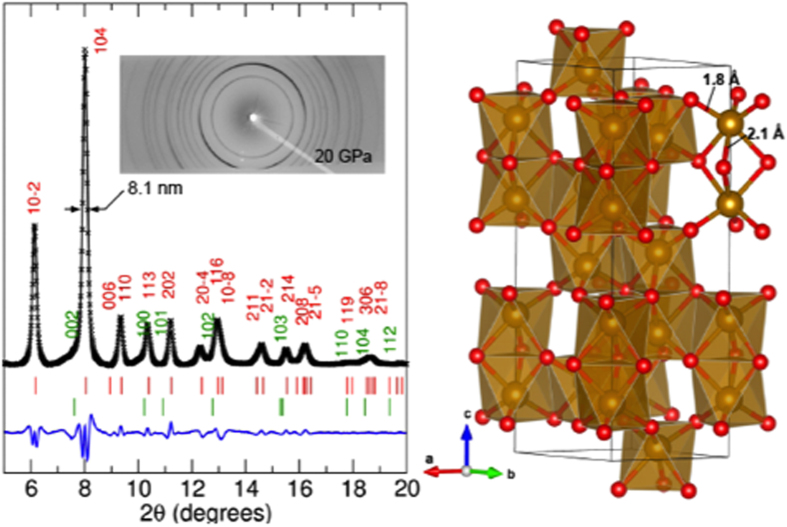
(**a**) Powder x-ray diffraction patterns of polymerized Fe(CO)_5_ at 20 GPa. Structure was fitted with trigonal structure with a hexagonal setting, *R-3c*, which is adapted from the hematite structure^31^. Cross symbols, black solid line, and red vertical tick marks presents observed, Riedvelt fit, and peak positions respectively. Blue solid line presents the difference between data and calculation. The green lines indicate the diffraction positions of amorphous carbon graphite in a hexagonal structure with the parameters, a = 2.407 Å and c = 5.620 Å. The inset shows the *2D* diffraction image as obtained in the MAR image plate detector. (**b**) Crystal model of Fe_2_O_3_ in CO-polymer at 20 GPa. Yellow and red colors signify Fe and O atoms, respectively.
