# Supplementary material for: Phase Diagram and Transformations of Iron Pentacarbonyl to nm Layered Hematite and Carbon-Oxygen Polymer under Pressure
Source: Sci Rep. 2015 Oct 12;5:15139. doi: 10.1038/srep15139 (PMC4601022; doi:10.1038/srep15139)
Supplement: Supplementary Information [file srep15139-s1.pdf]

# Supplementary Information

# for

# Phase Diagram and Transformations of Iron Pentacarbonyl to nm Layered Hematite and Carbon-Oxygen Polymer under Pressure

Young Jay Ryu, Minseob Kim and Choong-Shik Yoo*

**Table S1.** Raman spectra of $Fe(CO)_5$ phases at 0.3 (I), 1.5 (II) and 4.9 (III) at 300 K, in comparison with those previously ambient pressure data at 300K and 150K [30].

| Mode | Assignment | | Ref. 30 | | Present (300K) | | | |
|---|---|---|---|---|---|---|---|---|
| | | | STP | 150K - 1atm | STP | I at 0.3 GPa | II at 1.5 GPa | III at 4.9 GPa |
| | | | | *C≡O Stretching modes* | | | | |
| $\upsilon_1$ | $A'_{1eq}$ | C≡O | 2116 | 2117 | 2116 | 2116 | 2118 | 2131 |
| $\upsilon_2$ | $A'_{1ax}$ | C≡O | 2030 | 2022 | 2030 | 2021 | 2031 | 2044 |
| | ….. | ….. | | | | | | 2023 |
| $\upsilon_{10}$ | $E'_{eq}$ | C≡O | 1989 | 1999 | 1990 | 1998 | 1991 | 1996 |
| | | | | 1971 | | 1970 | 1979 | 1982 |
| | | | | *Fe-CO stretching and Fe-C≡O bending modes* | | | | |
| $2\upsilon_5$ | $(A'_{2eq})^2$ | Fe-CO | 754 | 758 | 755 | 754 | 764 | 774 |
| $\upsilon_{11}$ | $E'_{eq}$ | Fe-CO | 653 | 658 | ….. | 654 | 657 | 668 |
| $\upsilon_{12}$ | $E'_{ax}$ | Fe-CO | 559 | 562 | 556 | 557 | 560 | 565 |
| $\upsilon_{16}$ | $E''_{eq}$ | Fe-CO | 491 | 496 | ….. | | | |
| $\upsilon_{13}$ | $E'_{eq}$ | Fe-C≡O | 482 | 490 | 486 | 491 | 492 | 507 |
| $\upsilon_{17}$ | $E'_{ax}$ | Fe-C-O | 448 | 450 | 444 | 446 | 461 | 481 |
| $\upsilon_3$ | $A'1_{eq}$ | Fe-C≡O | 418 | 425 | 416 | 423 | 430 | 450 |
| | ….. | ….. | | | | | | 442 |
| $\upsilon_4$ | $A'_{1ax}$ | Fe-C≡O | 381 | 380 | 380 | 378 | 379 | 380 |
| | ….. | ….. | | | | | 163 | 240 |
| | ….. | ….. | | | | | 189 | 205 |
| | ….. | ….. | | | | | 188 | |
| | ….. | ….. | | | | | 163 | |
| | | | | *C-Fe-C bending modes* | | | | |
| $\upsilon_{18}$ | $E''_{ax}$ | C-Fe-C | | 132 | | 132 | 152 | 186 |
| $\upsilon_{14}$ | $E'_{eq}$ | C-Fe-C | 107 | 118 | 108 | 123 | 137 | |
| | ….. | ….. | | 113 | | 112 | 126 | 154 |
| | ….. | ….. | | | | | 104 | 135 |
| $\upsilon_{15}$ | $E'_{ax}$ | C-Fe-C | 64 | 73 | 67 | 75 | 84 | 116 |
| | | | | 57 | | | | |

**Table S2.** Crystal structure models for $Fe_2O_3$ hematite determined based on the powder x-ray diffraction pattern of reacted $Fe_2(CO)_5$ at 20 GPa, showing in comparison with the previously observed hematite structures.[35]

| | α-$Fe_2O_3$ (18.9 GPa)[35] | $Fe_2O_3$ (20.0 GPa) |
|---|---|---|
| Space group | R-3c | R-3c |
| Lattice parameter(Å) | | |
| a | 4.9666(4) | 4.573(1) |
| b | 4.9666(4) | 4.573(1) |
| c | 13.4251(16) | 14.352(6) |
| Volume (Å$^3$) | 286.794 | 260.11(10) |
| Density, r (g/cm$^3$) | 5.548 | 6.121 |
| Atomic position | | |
| Fe, 12c (0,0,z) | 0.3547(1) | 0.854(2) |
| O, 18e (x,0,1/4) | 0.3228(10) | 0.326(11) |
| Bond distance (Å) | | |
| Fe-O | 1.9054 | 1.789(2) |
| Fe-O2 | 2.1322 | 2.103(3) |
| Fe-Fe2 | 2.8112 | 2.957(2) |
| Bond angle ($^o$) | | |
| Fe-O-Fe | 120.90 | 127.11(33) |
| O-Fe-O | 102.36 | 96.56(14) |

**Table S3.** Summary of structure parameter of $Fe(CO)_5$ phase I at ~1.0 GPa and ambient temperature, comparing with the previously reported one at 90 K and ambient pressure.

| | $Fe(CO)_5$ (90K)[38] | $Fe(CO)_5$-I (1.5 GPa) |
|---|---|---|
| Space group | C2/c | C2/c |
| Lattice parameter (Å) | | |
| a | 11.807(1) | 11.892(3) |
| b | 6.821(2) | 6.8767(16) |
| c | 9.367(4) | 9.455(3) |
| β | 107.72(1) | 107.53(1) |
| Volume (Å$^3$) | 718.6 | 737.4(3) |
| Density, ρ (g/cm$^3$) | 1.811 | 1.765 |
| Atomic position | | |
| Fe (4e) | (0.0000, 0.1667, 0.2500) | (0.0000, 0.6665, 0.2500) |
| C1 (8f) | (0.0831, 0.3035, 0.4136) | (-0.0610, 0.7960, 0.1420) |
| C2 (8f) | (0.1294, 0.1640, 0.1828) | (0.1310, 0.6610, 0.1900) |
| C3 (4e) | (0.0000, -0.0981, 0.2500) | (0.0000, 0.4010, 0.2500) |
| O1 (8f) | (0.1354, 0.3912, 0.5158) | (-0.1240, 0.8880, -0.0020) |
| O2 (8f) | (0.2092, 0.1609, 0.1413) | (0.2210, 0.6691, 0.1620) |
| O3 (4e) | (0.0000, -0.2639, 0.2500) | (0.0000, 0.2427, 0.2500) |
| Bond distance (Å) | | |
| Fe-C1 | 1.7964 | 1.83(2) |
| Fe-C2 | 1.8044 | 1.81(2) |
| Fe-C3 | 1.7993 | 1.38(6) |
| C1-O1 | 1.1310 | 1.49(8) |
| C2-O2 | 1.1137 | 1.18(3) |
| C3-O3 | 1.1274 | 1.09(3) |
| Fe-Fe2 | 5.1631 | 5.2529 |
| Fe-Fe3 | 6.7706 | 6.4879 |
| Bond angle ($^o$) | | |
| C3-Fe-C3 | 117.524 | 99.91 |
| C1-Fe-C3 | 121.238 | 130.05 |
| C2-Fe-C2 | 178.92 | 177.61 |
